# Tafenoquine versus Primaquine to Prevent Relapse of *Plasmodium vivax* Malaria

**DOI:** 10.1056/NEJMoa1802537

**Published:** 2019-01-17

**Authors:** A. Llanos-Cuentas, M.V.G. Lacerda, T.T. Hien, I.D. Vélez, C. Namaik-larp, C.S. Chu, M.F. Villegas, F. Val, W.M. Monteiro, M.A.M. Brito, M.R.F. Costa, R. Chuquiyauri, M. Casapía, C.H. Nguyen, S. Aruachan, R. Papwijitsil, F.H. Nosten, G. Bancone, B. Angus, S. Duparc, G. Craig, V.M. Rousell, S.W. Jones, E. Hardaker, D.D. Clover, L. Kendall, K. Mohamed, G.C.K.W. Koh, V.M. Wilches, J.J. Breton, J.A. Green

## Abstract

**BACKGROUND:**

Tafenoquine, a single-dose therapy for *Plasmodium vivax* malaria, has been associated with relapse prevention through the clearance of *P. vivax* parasitemia and hypnozoites, termed “radical cure.”

**METHODS:**

We performed a phase 3, prospective, double-blind, double-dummy, randomized, controlled trial to compare tafenoquine with primaquine in terms of safety and efficacy. The trial was conducted at seven hospitals or clinics in Peru, Brazil, Colombia, Vietnam, and Thailand and involved patients with normal glucose-6-phosphate dehydrogenase (G6PD) enzyme activity and female patients with moderate G6PD enzyme deficiency; all patients had confirmed *P. vivax* parasitemia. The patients were randomly assigned, in a 2:1 ratio, to receive a single 300-mg dose of tafenoquine or 15 mg of primaquine once daily for 14 days (administered under supervision); all patients received a 3-day course of chloroquine and were followed for 180 days. The primary safety outcome was a protocol-defined decrease in the hemoglobin level (>3.0 g per deciliter or ≥30% from baseline or to a level of <6.0 g per deciliter). Freedom from recurrence of *P. vivax* parasitemia at 6 months was the primary efficacy outcome in a planned patient-level meta-analysis of the current trial and another phase 3 trial of tafenoquine and primaquine (per-protocol populations), and an odds ratio for recurrence of 1.45 (tafenoquine vs. primaquine) was used as a noninferiority margin.

**RESULTS:**

A protocol-defined decrease in the hemoglobin level occurred in 4 of 166 patients (2.4%; 95% confidence interval [CI], 0.9 to 6.0) in the tafenoquine group and in 1 of 85 patients (1.2%; 95% CI, 0.2 to 6.4) in the primaquine group, for a between-group difference of 1.2 percentage points (95% CI, −4.2 to 5.0). In the patient-level meta-analysis, the percentage of patients who were free from recurrence at 6 months was 67.0% (95% CI, 61.0 to 72.3) among the 426 patients in the tafenoquine group and 72.8% (95% CI, 65.6 to 78.8) among the 214 patients in the primaquine group. The efficacy of tafenoquine was not shown to be noninferior to that of primaquine (odds ratio for recurrence, 1.81; 95% CI, 0.82 to 3.96).

**CONCLUSIONS:**

Among patients with normal G6PD enzyme activity, the decline in hemoglobin level with tafenoquine did not differ significantly from that with primaquine. Tafenoquine showed efficacy for the radical cure of *P. vivax* malaria, although tafenoquine was not shown to be noninferior to primaquine. (Funded by GlaxoSmithKline and Medicines for Malaria Venture; GATHER ClinicalTrials.gov number, NCT02216123.)

*Plasmodium vivax* causes almost half the cases of malaria outside Africa.^1^ The *P. vivax* lifecycle includes a quiescent liver stage, the hypnozoite. Periodic reactivation of hypnozoites induces repeated clinical relapses. Hypnozoites are undetectable and constitute a silent transmission reservoir, challenging efforts to eliminate malaria.^2^ Clearance of *P. vivax* parasitemia and elimination of hypnozoites (termed “radical cure”) require combined treatment with primaquine (15 mg administered orally once daily for 14 days) and a schizonticide.^3^ Tafenoquine is a longer-acting antihypnozoite medication newly registered with the Food and Drug Administration and Australian Therapeutic Goods Administration for the radical cure of *P. vivax* malaria. Administered in a single dose of 300 mg in combination with a schizonticide, tafenoquine has a considerable advantage over the 14-day course of primaquine in terms of convenience.^4,5^

Both primaquine and tafenoquine cause drug-induced hemolysis in persons with glucose-6-phosphate dehydrogenase (G6PD) deficiency.^6-8^ This X-linked enzymopathy has a prevalence of 1 to 30% in populations at risk for *P. vivax* malaria.^9^ Currently available qualitative G6PD rapid tests use a threshold of G6PD enzyme activity of approximately 30% of normal (defined as the median value among healthy male volunteers) to classify persons as G6PD deficient. This threshold reliably identifies male hemizygotes and female homozygotes who have the G6PD-deficient gene and are at risk for severe hemolysis. However, according to these assays, female heterozygotes for the *G6PD* gene who have G6PD activity levels of 30 to 70% of normal may be identified as “G6PD normal” but remain at risk for hemolysis.^8,10,11^ The hemolytic risk with tafenoquine relative to that with primaquine has been characterized among healthy volunteers with normal G6PD enzyme activity and volunteers who were heterozygous for G6PD deficiency.^8^ However, a possible interaction between drug effects and malaria-induced hemolysis requires a formal evaluation of hemolytic risk among patients with *P. vivax* malaria.

The efficacy of a single dose of tafenoquine was assessed in two comparative trials (the Dose and Efficacy Trial Evaluating Chloroquine and Tafenoquine in Vivax Elimination [DETECTIVE] phase 2b and phase 3 trials), which showed that the risk of recurrent malaria at 6 months was approximately 70% lower with tafenoquine than with placebo.^4,5^ Historical data from clinical trials of primaquine show that the risk of recurrent malaria was approximately 60% lower with primaquine than with placebo,^12^ although no benefit from primaquine (administered by the patient without supervision) was noted.^12,13^ However, a direct comparison between tafenoquine and primaquine (administered under supervision) in terms of efficacy is critical for decision making by physicians.

Here, we report comparative data on the safety and efficacy of tafenoquine, as compared with primaquine, to attain a radical cure for *P. vivax* malaria. The Global Assessment of Tafenoquine Hemolytic Risk (GATHER) randomized, controlled trial aimed to compare hemolytic risk among patients with normal G6PD enzyme activity and female patients with moderate G6PD deficiency (≥40 to <70% of the median value of G6PD enzyme activity among healthy male volunteers with normal G6PD activity, as determined at each trial site [hereafter, the site-specific normal value]). The safety of tafenoquine and primaquine was also compared in an integrated safety analysis of the phase 2b and 3 DETECTIVE trials and the GATHER trial. Although primaquine was tested in these trials, they were not powered for comparisons of efficacy between tafenoquine and primaquine. Thus, a planned patient-level meta-analysis of the GATHER trial and phase 3 DETECTIVE trial was conducted with the use of a noninferiority design to evaluate freedom from recurrence of *P. vivax* parasitemia at 6 months with tafenoquine, as compared with primaquine.^5^

## METHODS

### TRIAL DESIGN AND OVERSIGHT

The GATHER trial was a phase 3, prospective, double-blind, double-dummy, randomized, controlled trial that was conducted at seven hospitals or clinics in Peru, Brazil, Colombia, Vietnam, and Thailand between April 30, 2015, and November 4, 2016. It was conducted in accordance with the International Conference on Harmonisation Good Clinical Practice guidelines, the tenets of the Declaration of Helsinki (2013), and relevant regulatory requirements. The ethics committee or institutional review board at each participating site provided ethical approval. All patients provided written informed consent or written assent with written consent from their parent or guardian.

The protocol, including five amendments, is available with the full text of this article at NEJM.org. The sponsors of the trial (GlaxoSmithKline and Medicines for Malaria Venture) contributed to the development of the protocol. GlaxoSmith-Kline managed the conduct of the trial, collected and managed the data, monitored the trial staff, conducted the statistical analysis, and provided all the drugs used in the trial for initial treatment. The authors vouch for the accuracy and completeness of the data and for the fidelity of the trial to the protocol. The methods were similar to those used in the phase 3 DETECTIVE trial^5^ and are outlined briefly here.

### PATIENTS

Patients were eligible for enrollment in the trial if they were at least 16 years of age and had microscopically confirmed *P. vivax* infection (>100 and <100,000 parasites per microliter). Other inclusion criteria were similar to the criteria in the phase 3 DETECTIVE trial,^5^ except that female patients were required to have a G6PD enzyme level that was at least 40% of the site-specific normal value and male patients were required to have a G6PD enzyme level that was at least 70% of the site-specific normal value. Patients were required to have a hemoglobin level of at least 7 g per deciliter, except for the female patients with moderate G6PD enzyme activity, who were required to have a hemoglobin level of at least 8 g per deciliter.

### RANDOMIZATION AND TREATMENTS

The trial drugs were chloroquine phosphate (300-mg tablets [West Ward Pharmaceuticals] or 150-mg tablets [Alliance Pharmaceuticals]), tafenoquine (150-mg film-coated tablets [GlaxoSmithKline]), and primaquine phosphate (15-mg overencapsulated tablets [Sanofi]) (dosages are given for the free-base form). All patients received 600 mg of open-label chloroquine on days 1 and 2 and 300 mg on day 3. Patients were randomly assigned, in a 2:1 ratio, to receive a single 300-mg dose of tafenoquine while hospitalized on day 1 or 2 or 15 mg of primaquine once daily for 14 days starting on day 1 or 2. All treatments were administered orally with food. To ensure that the patients and investigators were unaware of the trial-group assignments, primaquine-matched placebo was administered to patients in the tafenoquine group and tafenoquine-matched placebo was administered to patients in the primaquine group on the scheduled treatment days. Treatment was discontinued when a patient had a protocol-defined decrease in the hemoglobin level (>3.0 g per deciliter or ≥30% from baseline or to a level of <6.0 g per deciliter), clinically significant changes in the liver chemical profile, a prolongation of the QT interval (corrected with the use of Fridericia’s formula) to more than 500 msec, or a grade 4 adverse event (Table S1 in the Supplementary Appendix, available at NEJM.org).

### PROCEDURES

Trial patients were hospitalized on days 1 to 3, and the trial visits took place on days 5, 8, 11, and 15 during the treatment period and on days 22, 29, 60, 90, 120, 150, and 180 during the follow-up period. Safety, clinical, laboratory, and parasitologic assessments were consistent with those in the phase 3 DETECTIVE trial.^5^ *P. vivax* genotyping and cytochrome P450 2D6 (CYP2D6) genotyping were performed as described in the phase 3 DETECTIVE trial.^5,14-18^ The incidence of genetically homologous or heterologous *P. vivax* infection was assessed with the use of previously published markers.^15^ G6PD phenotype was determined by means of quantitative spectrophoto-metric analysis (Trinity Biotech).^5^ In addition, a qualitative G6PD test (CareStart, AccessBio) was performed at screening, but the findings were not used to determine eligibility. G6PD genotype was determined for all female patients and for male patients who had a protocol-defined decrease in the hemoglobin level during the treatment period.^19,20^

### TRIAL POPULATIONS AND OUTCOMES

The safety population included all patients who underwent randomization and received at least one dose of a trial medication in a blinded manner. The intention-to-treat population was a subgroup of patients from the safety population who had microscopically confirmed *P. vivax* parasitemia. The per-protocol population was a subgroup of patients from the intention-to-treat population who had no major protocol violations.

In the GATHER trial, the primary safety outcome was a protocol-defined decrease in the hemoglobin level (>3.0 g per deciliter or ≥30% from baseline or to a level of <6.0 g per deciliter). Secondary safety outcomes were the occurrence and severity of adverse events and the occurrence of abnormal clinical laboratory test results, electrocardiograms, vital signs, or findings from ophthalmic assessments. The safety of tafenoquine and primaquine was also compared in an integrated safety analysis that included data from the safety populations in the GATHER trial and phase 2b and 3 DETECTIVE trials. Secondary efficacy outcomes in the GATHER trial were freedom from recurrence of *P. vivax* parasitemia at 4 months and 6 months and other outcomes consistent with those in the phase 3 DETECTIVE trial and were evaluated in the per-protocol and intention-to-treat populations.^5^

The primary efficacy outcome of freedom from recurrence of *P. vivax* parasitemia at 6 months was evaluated in a planned patient-level meta-analysis of the per-protocol populations in the GATHER trial and phase 3 DETECTIVE trial. Freedom from recurrence was defined as initial clearance of parasitemia (parasite numbers decreased to below the limit of detection in a thick blood smear and remained undetectable in a second smear collected 6 to 12 hours later), without the patient presenting with *P. vivax* asexual stage parasites at any point in the trial, plus a negative *P. vivax* smear within the acceptable time window for the 6-month assessment.

### STATISTICAL ANALYSIS

A sample size of 300 patients — 250 male and female patients with normal G6PD enzyme activity and 50 female patients with moderate G6PD enzyme deficiency (>40 to <70% of the site-specific normal value) — was planned in the GATHER trial. The sample size was determined on the basis of a regulatory requirement to obtain an appropriate overall safety database for tafenoquine (300 mg) in patients with *P. vivax* malaria who had normal or moderately deficient G6PD activity. Analyses were performed with the use of SAS software, version 9.4 (SAS Institute). All safety data from the GATHER trial and from the integrated safety analysis of the GATHER trial and phase 2b and 3 DETECTIVE trials were summarized with the use of descriptive statistics. For the primary safety outcome in the GATHER trial, the percentage of patients who had a protocol-defined decrease in the hemoglobin level in each treatment group was reported with 95% confidence intervals, and the absolute difference in this percentage between the treatment groups was reported with 95% confidence intervals.

In the GATHER trial, the percentages of patients who were free from recurrence at 4 months and 6 months and the times to clearance of parasites, gametocytes, and fever were estimated with the use of the Kaplan–Meier method, as described in the phase 3 DETECTIVE trial.^5^ All other outcomes were summarized with the use of descriptive statistics.

In the patient-level meta-analysis, noninferiority of tafenoquine to primaquine with respect to freedom from recurrence at 6 months was assessed in the per-protocol populations in the GATHER trial and phase 3 DETECTIVE trial. The noninferiority margins were derived from the phase 2b DETECTIVE trial with the use of a 75% preserved effect of primaquine.^4^ The noninferiority margin was a hazard ratio for recurrence of 1.21, or if the assumption of proportional hazards was not met, an odds ratio was to be calculated. The odds ratio comparing chloroquine to primaquine in the phase 2b DETECTIVE trial, with adjustment for region, was 8.66 (95% confidence interval [CI], 2.80 to 26.85). The non-inferiority margin of 1.45 used in the meta-analysis was derived from taking the 75% preserved effect of the lower 95% confidence bound of 2.80. The final analysis was based on an odds ratio from a logistic-regression model that was adjusted for trial, region, and interaction between treatment and region. A sensitivity analysis was performed in the intention-to-treat population. As a consequence of a significant interaction between treatment and region, a post hoc sensitivity analysis was performed, in which the data from Southeast Asia in the phase 3 DETECTIVE trial were excluded. A post hoc difference in risk was determined with the use of the Mantel– Haenszel method, with stratification according to trial and region.

## RESULTS

### PATIENTS

**Figure 1:**
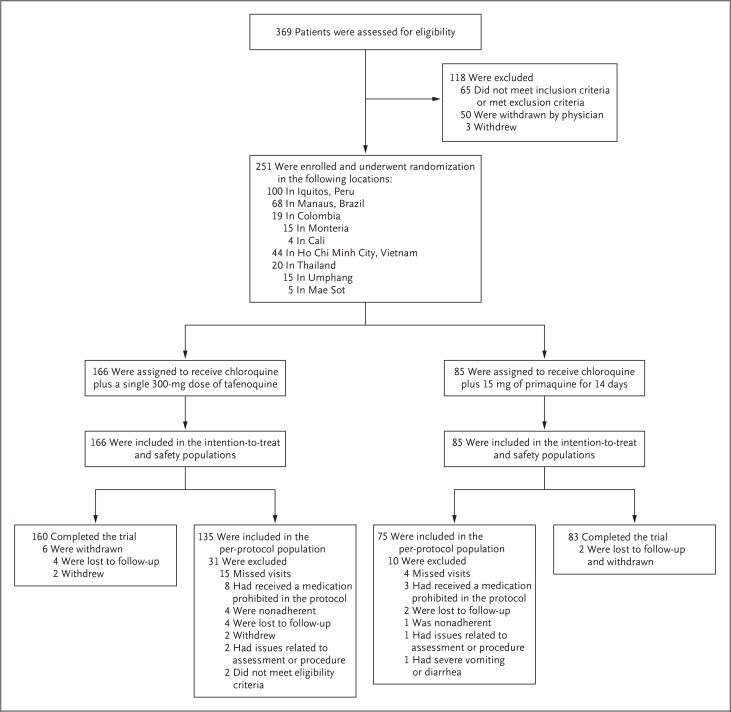
**Enrollment, Randomization, and Trial Populations in the GATHER Trial**. The safety population included all patients who underwent randomization and received at least one dose of a trial medication in a blinded manner. The intention-to-treat population was a subgroup of patients from the safety population who had microscopically confirmed *Plasmodium vivax* parasitemia. The per-protocol population was a subgroup of patients from the intention-to-treat population who had no major protocol violations. Patients may have had more than one reason for exclusion from the analysis populations. GATHER denotes Global Assessment of Tafenoquine Hemolytic Risk.

A total of 251 patients were recruited in the GATHER trial; 74.5% were from South America (Fig. 1 and Table 1). The qualitative G6PD point-of-care test, as compared with the quantitative assay, identified 1 of 6 patients with G6PD enzyme levels lower than 70% of the site-specific median value as “normal.” A total of 82 of 83 patients (98.8%; 2 patients were lost to follow-up) in the primaquine group met the criteria for adherence to primaquine (≥12 doses taken, as assessed by pill count and measurement of blood levels of primaquine and its metabolite 7-carboxyprimaquine on days 8 and 15). The integrated safety analysis included 483 patients who received tafenoquine and 264 patients who received primaquine (Tables S2 through S4 in the Supplementary Appendix).

**Table 1:** Baseline Demographic and Clinical Characteristics of the Patients in the GATHER Trial (Intention-to-Treat and Safety Populations).*

Characteristic	Tafenoquine (N = 166)	Primaquine (N = 85)
Male sex — no. (%)	114 (68.7)	53 (62.4)
Age — yr	37.5±14.3	37.7±14.7
Body weight — kg	65.5±14.1	63.7±11.0
Region — no. (%)		
South America	125 (75.3)	62 (72.9)
Southeast Asia	41 (24.7)	23 (27.1)
Race or ethnic group — no. (%)†		
American Indian	87 (52.4)	43 (50.6)
Asian	41 (24.7)	23 (27.1)
Multiple	36 (21.7)	19 (22.4)
Black	2 (1.2)	0
Median no. of *P. vivax* asexual forms (range) per *μ*l	3618 (102–45,410)	5079 (104–82,650)
Median no. of *P. vivax* gametocytes (range) per *μ*l	45 (0–2015)	60 (0–5340)
Previous malaria — no. (%)		
Yes	132 (79.5)	63 (74.1)
No	32 (19.3)	22 (25.9)
Unknown	2 (1.2)	0
G6PD enzyme activity — IU/g of hemoglobin		
Mean	8.3±1.3	8.2±1.3
Range	6.0–13.5	5.1–14.2
G6PD enzyme activity — % of site-specific normal value‡		
Mean	98.8±15.2	97.4±16.2
Range	70.1–170.5	62.0–169.2
CYP2D6 metabolic activity phenotype — no. (%)§		
Poor	1 (0.6)	0
Intermediate	31 (18.7)	19 (22.4)
Extensive	111 (66.9)	51 (60.0)
Unknown	24 (14.5)	15 (17.6)

* Plus–minus values are means ±SD. Percentages may not total 100 because of rounding. There were no clinically meaningful differences in demographic and clinical characteristics at baseline between the two treatment groups. The safety population included all patients who underwent randomization and received at least one dose of a trial medication in a blinded manner. The intention-to-treat population was a subgroup of patients from the safety population who had microscopically confirmed *Plasmodium vivax* parasitemia. GATHER denotes Global Assessment of Tafenoquine Hemolytic Risk.

† Race or ethnic group was reported by the patient.

‡ The site-specific normal value was the median value of glucose-6-phosphate dehydrogenase (G6PD) enzyme activity among healthy male volunteers with normal G6PD activity, as determined at each trial site.

§ Cytochrome P450 2D6 (CYP2D6) metabolic activity phenotype was determined according to the Activity Score system (an activity score of 0 indicates poor metabolic activity, a score of 0.5 or 1 indicates intermediate activity, and a score of 1.5 or higher indicates extensive activity), modified from Gaedigk et al.^21^

In the patient-level meta-analysis of the GATHER trial the total per-protocol population included 327 patients in the tafenoquine group (76.8% of the patients who were assigned to receive tafenoquine in the two trials) and 183 patients in the primaquine group (85.5% of the patients who were assigned to receive primaquine in the two trials). Demographic and clinical characteristics at baseline were similar in the two treatment groups, with no clinically meaningful betweengroup differences. A total of 181 of 183 patients (98.9%) in the primaquine group met the criteria for adherence to primaquine (described above). Additional details about the per-protocol population in the meta-analysis are provided in Tables S5 through S7 in the Supplementary Appendix.

### HEMATOLOGIC SAFETY

**Figure 2:**
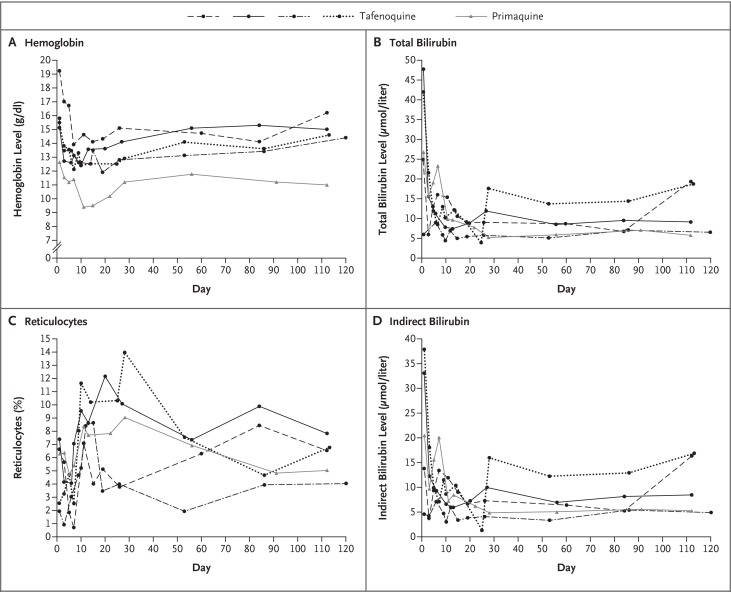
**Changes in Key Hematologic Measures among Individual Patients Who Met the Primary Safety Outcome of a Protocol-Defined Decrease in the Hemoglobin Level in the GATHER Trial (Safety Population)**. A protocol-defined decrease in the hemoglobin level (>3.0 g per deciliter or ≥30% from baseline or to a level of <6.0 g per deciliter) occurred in 4 of 166 patients in the tafenoquine group and in 1 of 85 patients in the primaquine group. To convert bilirubin to milligrams per deciliter, divide by 17.1.

In the GATHER trial, the primary outcome of a protocol-defined decrease in the hemoglobin level occurred in 4 of 166 patients (2.4%; 95% CI, 0.9 to 6.0) in the tafenoquine group and in 1 of 85 patients (1.2%; 95% CI, 0.2 to 6.4) in the prima-quine group, for a between-group difference of 1.2 percentage points (95% CI, −4.2 to 5.0) (Fig. 2). All primary-outcome events occurred in male patients who had a normal G6PD genotype and phenotype and did not require clinical intervention (Table S8 in the Supplementary Appendix). The extent of changes in mean hemoglobin and hematocrit levels and their time courses, as well as the mean changes from baseline in hemoglobin levels, were similar in the treatment groups, with a slight decline followed by recovery by day 60 (Figs. S1 through S3 in the Supplementary Appendix). A protocol-defined decrease in the hemoglobin level could not be evaluated in G6PD heterozygous female patients because only 1 was recruited. This woman was recruited from Umphang, Thailand, and had the *G6PD Mahidol* variant and moderate G6PD enzyme deficiency (5.1 IU per gram of hemoglobin [62% of the site-specific normal value]). In addition, a heterozygous woman who had a *G6PD* variant of unknown significance was recruited from Ho Chi Minh City, Vietnam (normal G6PD enzyme activity of 8.8 IU per gram of hemoglobin [105% of the site-specific normal value]). Both women received primaquine and had no protocol-defined decrease in the hemoglobin level.

In the integrated safety analysis, tafenoquine and primaquine had similar hemoglobin profiles (Fig. S4 in the Supplementary Appendix). In the tafenoquine group, the lowest mean (±SD) hemoglobin level occurred on day 3 (11.5±1.3 g per deciliter among the female patients and 12.9±1.5 g per deciliter among the male patients), whereas in primaquine group, the lowest mean hemoglobin level occurred on day 15 among the female patients (11.5±1.0 g per deciliter) and on day 3 among the male patients (13.4±1.6 g per deciliter).

No patient had a hemoglobin level lower than 7.3 g per deciliter in the tafenoquine group or lower than 6.7 g per deciliter in the primaquine group, and no patient had an intervention for a decrease in the hemoglobin level; in all cases of a decline in hemoglobin level, the level returned spontaneously to the baseline value.

The methemoglobin level increased in both treatment groups, with a greater increase in the primaquine group, but the patients had no clinical symptoms. No other hematologic outcome differed notably between the treatment groups. Additional details are provided in Figure S5 and Tables S9 and S10 in the Supplementary Appendix.

### SAFETY

In the GATHER trial, the percentage of patients with adverse events up to day 29 was similar in the tafenoquine group (54.8% [91 of 166 patients]) and in the primaquine group (50.6% [43 of 85 patients]) (Table 2). Two serious adverse events occurred in the tafenoquine group (1 patient had pyrexia and 1 had pneumonia); neither event was attributed to a trial medication by the site investigators, who were unaware of the treatment-group assignments.

**Table 2:** Most Common Adverse Events of Any Cause That Occurred from the Start of Treatment through Day 29 (Safety Population).*

Event	GATHER Trial		Integrated Safety Analysis	
	Tafenoquine (N = 166)	Primaquine (N = 85)	Tafenoquine (N = 483)	Primaquine (N = 264)
	*number of patients (percent)*			
Any adverse event	91 (54.8)	43 (50.6)	251 (52.0)	131 (49.6)
Pruritus	20 (12.0)	19 (22.4)	57 (11.8)	36 (13.6)
Dizziness	27 (16.3)	13 (15.3)	52 (10.8)	23 (8.7)
Nausea	16 (9.6)	6 (7.1)	36 (7.5)	14 (5.3)
Headache	19 (11.4)	10 (11.8)	34 (7.0)	19 (7.2)
Vomiting	11 (6.6)	5 (5.9)	28 (5.8)	15 (5.7)
Abdominal pain, upper	8 (4.8)	1 (1.2)	21 (4.3)	13 (4.9)
Diarrhea	6 (3.6)	3 (3.5)	19 (3.9)	9 (3.4)
Hemoglobin decreased	4 (2.4)	1 (1.2)	19 (3.9)	4 (1.5)
Insomnia	2 (1.2)	0	14 (2.9)	8 (3.0)
Urinary tract infection	6 (3.6)	3 (3.5)	14 (2.9)	6 (2.3)
Back pain	3 (1.8)	2 (2.4)	12 (2.5)	3 (1.1)
Creatine phosphokinase increased	2 (1.2)	1 (1.2)	10 (2.1)	2 (0.8)
Nasopharyngitis	6 (3.6)	2 (2.4)	9 (1.9)	5 (1.9)
Alanine aminotransferase increased	0	0	8 (1.7)	6 (2.3)
Asthenia	4 (2.4)	4 (4.7)	7 (1.4)	4 (1.5)
Pharyngitis	2 (1.2)	2 (2.4)	7 (1.4)	6 (2.3)
Pyrexia	3 (1.8)	1 (1.2)	5 (1.0)	3 (1.1)
Vision blurred	0	0	5 (1.0)	3 (1.1)
Cough	1 (0.6)	1 (1.2)	4 (0.8)	3 (1.1)
Electrocardiogram QT prolonged	0	0	3 (0.6)	5 (1.9)
Hypokalemia	0	0	2 (0.4)	3 (1.1)
Methemoglobinemia	0	0	0	3 (1.1)

* Shown are the adverse events that occurred in more than 1% of the patients in either treatment group in the integrated safety analysis; adverse events are listed in the order of frequency in the tafenoquine group in the integrated safety analysis. Safety data through day 29 are presented to avoid the potential confounding effects of retreatment with primaquine plus chloroquine in the event of patients having recurrence. Details of all adverse events occurring at any point during the 6-month trial period and their severity grade are included in Table S11 in the Supplementary Appendix. Adverse events were classified according to the *Medical Dictionary for Regulatory Activities*, version 19.1. The integrated safety analysis included data from the safety populations in the phase 2b and 3 Dose and Efficacy Trial Evaluating Chloroquine and Tafenoquine in Vivax Elimination (DETECTIVE) trials and the GATHER trial.

In the integrated safety analysis, the percentage of patients who had a decrease in the hemoglobin level was higher in the tafenoquine group (3.9% [19 of 483 patients]) than in the prima-quine group (1.5% [4 of 264 patients]) (Table 2). Across the 6-month trial period, most adverse events were mild to moderate in severity; a grade 1 or 2 event occurred in 310 of the 321 patients (96.6%) in the tafenoquine group who had an adverse event and in 162 of the 172 patients (94.2%) in the primaquine group who had an adverse event. The percentage of patients with serious adverse events was similar in the tafenoquine group (6.0% [29 of 483 patients]) and in the primaquine group (4.5% [12 of 264 patients]). All adverse events resolved spontaneously, and none led to a discontinuation of treatment. Additional details on adverse events in the integrated safety analysis are provided in Tables S11 and S12 in the Supplementary Appendix.

Laboratory assessments showed an increase in mean creatinine levels on day 5 in the tafenoquine group.^22^ Slight, transient asymptomatic elevations in alanine aminotransferase levels occurred in both treatment groups. No other laboratory finding differed notably between the treatment groups. Additional details on laboratory safety assessments are provided in Tables S13 through S15 in the Supplementary Appendix.

On electrocardiographic evaluation, a slight, asymptomatic prolongation of the corrected QT interval (calculated with the use of Fridericia’s formula) was observed in both treatment groups — a finding that is consistent with the known effect of chloroquine (Table S16 in the Supplementary Appendix). Postbaseline abnormal findings on ophthalmic assessments were uncommon in the combined safety populations in all three trials: among the patients in the tafenoquine group, 1 of 107 (0.9%) had vortex keratopathy, 1 of 102 (1.0%) had retinal hyperpigmentation, and 1 of 102 (1.0%) had retinal hypopigmentation (retinal examination was not performed in 5 patients), and among the patients in the prima-quine group, 1 of 56 (1.8%) had retinal hypopigmentation (retinal examination was not performed in 1 patient) (Table S17 in the Supplementary Appendix).

### EFFICACY

In the GATHER trial, efficacy was prespecified as a secondary outcome. The percentage of patients who were free from recurrence at 6 months in the per-protocol population was 74.1% (100 of 135 patients) in the tafenoquine group and 76.0% (57 of 75 patients) in the primaquine group. Kaplan–Meier estimates of freedom from recurrence at 6 months in the per-protocol population were 73.3% (95% CI, 64.7 to 80.2) in the tafenoquine group and 76.0% (95% CI, 64.6 to 84.1) in the primaquine group; the corresponding values in the intention-to-treat population were 72.7% (95% CI, 64.8 to 79.2) and 75.1% (95% CI, 64.2 to 83.2) (Fig. S6 and Table S18 in the Supplementary Appendix).

The findings from a categorical analysis in which patients with missing data were considered to have had recurrence of *P. vivax* parasitemia were consistent with the above findings; the percentages of patients who were free from recurrence at 6 months were 67.5% (112 of 166 patients) in the tafenoquine group and 70.6% (60 of 85 patients) in the primaquine group. The other efficacy results were consistent with those from the phase 3 DETECTIVE trial, except that in the GATHER trial, CYP2D6 metabolizer class did not appear to affect efficacy outcomes in either treatment group, but only 1 patient with poor CYP2D6 metabolic activity was recruited. Additional details are provided in Tables S19 through S24 in the Supplementary Appendix.

### EFFICACY META-ANALYSIS

**Figure 3:**
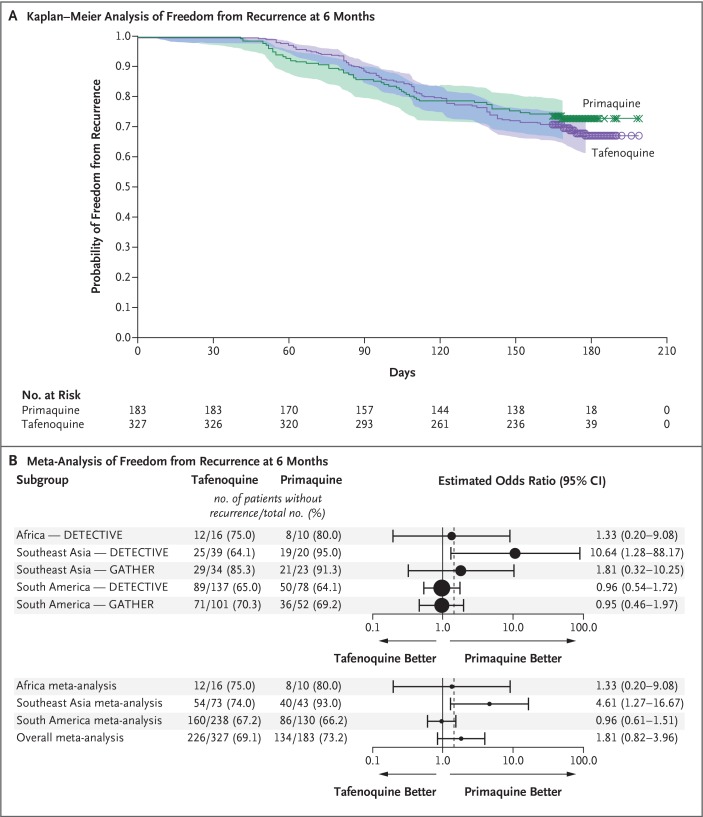
**Patient-Level Meta-Analysis of the Primary Efficacy Outcome of Freedom from Recurrence of *P. vivax* Parasitemia at 6 Months (Per-Protocol Population)**. Panel A shows the Kaplan–Meier analysis of freedom from recurrence of *P. vivax* parasitemia at 6 months in the tafenoquine group, as compared with the primaquine group, across the phase 3 Dose and Efficacy Trial Evaluating Chloroquine and Tafenoquine in Vivax Elimination (DETECTIVE) trial and the GATHER trial. Censored data are indicated by X’s in the primaquine group and by open circles in the tafenoquine group. Panel B shows the estimated odds ratios according to trial and region (top; the size of the solid circles indicates the study size) and the results from the meta-analysis of treatment difference in freedom from recurrence at 6 months across the phase 3 DETECTIVE trial and the GATHER trial (bottom). The dashed vertical line represents the prespecified noninferiority margin of an odds ratio for recurrence of 1.45 (tafenoquine vs. primaquine), which was derived from the phase 2b DETECTIVE trial.

The Kaplan–Meier estimates for the primary outcome of freedom from recurrence at 6 months in the per-protocol population in the meta-analysis were 67.0% (95% CI, 61.0 to 72.3) in the tafenoquine group and 72.8% (95% CI, 65.6 to 78.8) in the primaquine group (Fig. 3A, and Table S25 in the Supplementary Appendix). With respect to the primary outcome, noninferiority of tafenoquine to primaquine could not be shown (odds ratio for recurrence [tafenoquine vs. prima-quine], 1.81 [95% CI, 0.82 to 3.96]), although the confidence intervals were wide (Fig. 3B, and Table S26 in the Supplementary Appendix).

There was a significant interaction between treatment and region that resulted from an unexpectedly higher percentage of patients in Southeast Asia who were free from recurrence at 6 months with primaquine than with tafenoquine in the phase 3 DETECTIVE trial, and an ad hoc analysis that excluded this stratum showed an odds ratio for recurrence of 1.02 (95% CI, 0.66 to 1.57). The results in the intention-to-treat population were consistent with those in the per-protocol population. The risk difference between tafenoquine and primaquine was 4 percentage points (95% CI, –4 to 12) in favor of primaquine. Additional details are provided in Figure S7 and Tables S25 through S28 in the Supplementary Appendix.

## DISCUSSION

This report compares tafenoquine with prima-quine (administered under supervision) in terms of safety and efficacy for the radical cure of *P. vivax* malaria. The safety profile of the two agents was similar — both caused slight declines in hemoglobin level among patients with normal G6PD enzyme activity, and the between-group differences in the incidence and severity of the declines were relatively small. Among healthy patients with normal G6PD enzyme activity, neither tafenoquine nor primaquine caused a decline in hemoglobin level greater than 1.9 g per deciliter from baseline,^8^ and the observed declines in hemoglobin level in the GATHER trial were consistent with malaria and rehydration.^23^ The evaluation of hemolytic safety in the GATHER trial was limited, because female heterozygotes who had moderate G6PD enzyme deficiency could not be sufficiently recruited; only one such person met the study inclusion criteria despite an extension of the study recruitment window. In a study involving healthy volunteers with G6PD deficiency (G6PD enzyme activity of 40 to 60% of the site-specific normal value) who were heterozygous for the *Mahidol* ^487A^ G6PD-deficient variant, declines in hemoglobin level were self-limited and were similar in patients who received tafenoquine (−2.65 to −2.95 g per deciliter [three patients]) and in those who received primaquine (−1.25 to −3.0 g per deciliter [five patients]).^8^ However, the hemolytic safety of tafenoquine still requires evaluation among patients with *P. vivax* malaria who have G6PD enzyme activity less than 70% of the site-specific normal value.

In the meta-analysis, the majority of patients remained free from recurrence of *P. vivax* parasitemia at 6 months — 69.1% (226 of 327 patients) in the tafenoquine group and 73.2% (134 of 183 patients) in the primaquine group. The noninferiority of tafenoquine to primaquine could not be shown.^24^ The observed variability across regions added to the limitations of the analysis on the basis of just two studies. Although we did not meet our prespecified noninferiority margin, the between-group difference of 4 percentage points may be within the range of acceptable clinical variability.^24^ Furthermore, in the phase 3 DETECTIVE trial, the percentage of patients who were free from recurrence at 6 months in Southeast Asia was unexpectedly higher with prima-quine than with tafenoquine (91.5% [95% CI, 70.0 to 97.8] among 26 patients vs. 59.4% [95% CI, 43.8, 71.9] among 50 patients).^5^ These data were inconsistent with the findings from the phase 2b DETECTIVE trial^4^ and the GATHER trial, which showed that this efficacy outcome was similar in the two treatment groups in Southeast Asia. Moreover, a larger study at the same site in Cambodia showed that after a 14-day regimen of 30 mg of primaquine daily, 44 of 72 patients (61.1%; 95% CI, 50 to 72) were free from recurrence of *P. vivax* parasitemia at 6 months.^25^ The effect of the higher percentage of patients in Southeast Asia who met the efficacy outcome with primaquine than with tafenoquine in the phase 3 DETECTIVE trial was evident when these data were excluded from the analysis; after exclusion, the odds ratio for recurrence (tafenoquine vs. primaquine) was close to unity (1.02; 95% CI, 0.66 to 1.57).

Despite the known benefits of antirelapse therapy with primaquine, most patients with *P. vivax* malaria receive only a schizonticide.^26^ The current trial and the combined analysis from this trial and the phase 2b and phase 3 DETECTIVE trials showed that a single dose of tafenoquine can be administered without safety concerns in patients with *P. vivax* malaria who have normal G6PD enzyme activity. Although there was a possibility that primaquine offered a slight efficacy advantage over tafenoquine in Southeast Asia in this highly adherent population, extensive resources were deployed to support adherence to primaquine. Without such interventions, adherence to primaquine has been reported to be as low as 24% in Southeast Asia, with a corresponding attenuation of efficacy.^27,28^ In contrast, there are operational advantages to a single-dose medication, such as tafenoquine. The convergence of enhanced G6PD testing technology^26^ and the convenience of single-dose tafenoquine present an opportunity to further examine ways to attain an effective radical cure for *P. vivax* malaria.

## Notes

A data sharing statement provided by the authors is available with the full text of this article at NEJM.org.

Presented at the 6th International Conference on *Plasmodium vivax* Research (ICPVR), Manaus, Brazil, June 11–14, 2017; and at the 66th Annual Meeting of the American Society of Tropical Medicine and Hygiene, Baltimore, November 5–9, 2017.

Supported by GlaxoSmithKline (GSK) and Medicines for Malaria Venture. The meta-analysis was funded and conducted by GSK.

Disclosure forms provided by the authors are available with the full text of this article at NEJM.org.

We thank the site staff, the patients, and other persons who participated in the conduct of the study. We also thank the following trial staff: Sonia Torres, Ricardo Panduro, Piere Arevalo, Cesar Banda, Silvia Marin, Jorge Parraguez, Jhon Cochaches, and Lilia Pinedo (Iquitos, Peru); Monica Santos (Manaus, Brazil); the director, board, and staff at Phuoc-Long Hospital, Binh Phuoc Province (Phuoc-Long, Vietnam); the director, board, and staff at Bac-Ai Medical Center, Ninh Thuan Province (Bac-Ai, Vietnam); Lina Salazar, Adriana Alvarez, Efrén Quinterio, and Giacomo Salazar (Montería, Colombia); Worawit Tontiwattanasap, Khanitha Tarawa, Kanjana Keereepinit, Wachirada Pitiya, and Wanlee Khunyosying (Umphang, Thailand); Stéphane Proux, Lily Keereecharoen, and Kanlaya Sriprawat (Mae Sot, Thailand); staff at the Centro de Investigaciones Caucaseco and the Insalpa Buena-ventura, Mauricio Gonzales, Marta Lucia Ceballos, Claudia Gonzalez, Deyanira Sanchez, and Rafael Simancas (Cali, Colombia); Françoise Brand and Hans-Peter Beck (Swiss Tropical and Public Health Institute and University of Basel, Basel, Switzerland); Alison Webster (GSK clinical); Kim Fletcher and Cletus Ugwuegbulam (GSK operations); Deborah Kelly, Jessica Ackert, and Renata Buckley (GSK safety); Richard Ward and Terry Ernest (GSK manufacturing and supply); Nick Carter, Andrew Mastromarino, Jenny Scott, Thomas Drury, and Chris Nystrom (GSK statistics and programming); Navinkumar Goyal (GSK clinical pharmacology); Maxine Taylor (GSK drug disposition); Robert Stocken and Christian Baumann (GSK regulatory); Maggie Lynch (GSK project management); Isabelle Borghini-Fuhrer and Wiweka Kaszubska (Medicines for Malaria Venture); and Janice Culpepper (Bill and Melinda Gates Foundation). We acknowledge the following persons for in-country study management and monitoring: Natasha Dsouza, Nushara Nidhipaichit, Tanyada Yongchaitrakul, Filippi Bossatto, Carolina Seixas, Vivian Aboud, Evelyn Eder, and Rafael Paletta (GSK); and Tasaneeya Supaperm, Ruethai Wongchai, Salisa Pohpeera, Numfon Russamesuk, Carmen Rojas Vidal, Juan Vargas Valladares, Jesus Ramirez Morales, Murtaza Shipchandler, and Van Anh Hoang (Pharmaceutical Product Development). We acknowledge the following persons for additional support (funded by GSK): Alex Lowe of Fishawack Indicia, who provided administrative support for reviews of earlier versions of the manuscript, and Naomi Richardson of Magenta Communications, who developed the first draft of the manuscript, collated author contributions into subsequent drafts, and provided editorial, graphic, and proofreading support.

## APPENDIX

The authors’ full names and academic degrees are as follows: Alejandro Llanos-Cuentas, M.D., Marcus V.G. Lacerda, M.D., Tran T. Hien, M.D., Iván D. Vélez, Ph.D., Chayadol Namaik-larp, M.D., Cindy S. Chu, M.D., Maria F. Villegas, M.D., Fernando Val, Ph.D., Wuelton M. Monteiro, Ph.D., Marcelo A.M. Brito, M.D., Mônica R.F. Costa, M.D., Raul Chuquiyauri, M.D., Martín Casapía, M.D., Chau H. Nguyen, M.D., Sandra Aruachan, M.D., Ratchadaporn Papwijitsil, M.D., François H. Nosten, M.D., Germana Bancone, Ph.D., Brian Angus, M.D., Stephan Duparc, M.D., Graham Craig, B.Sc., Victoria M. Rousell, B.Sc., Siôn W. Jones, Ph.D., Elizabeth Hardaker, M.D., Donna D. Clover, B.Sc., Lindsay Kendall, M.Sc., Khadeeja Mohamed, M.Sc., Gavin C.K.W. Koh, Ph.D., Viviana M. Wilches, M.Biotech., John J. Breton, M.C.M., and Justin A. Green, M.D.

The authors’ affiliations are as follows: Universidad Peruana Cayetano Heredia, Lima, Peru (A.L.-C., R.C., M.C.); Fundação de Medicina Tropical Dr Heitor Vieira Dourado, Manaus (M.V.G.L., F.V., W.M.M., M.A.M.B., M.R.F.C.), and Fundação Oswaldo Cruz, Manguinhos, Rio de Janeiro (M.V.G.L.) — both in Brazil; Oxford University Clinical Research Unit, Ho Chi Minh City, Vietnam (T.T.H., C.H.N.); Universidad de Antioquia, Medellin (I.D.V.), Centro de Investigaciones Clinicas S.A.S de Cali, Cali (M.F.V.), and IMAT Oncomedica, Monteria (S.A.) — all in Colombia; Umphang Hospital, Tak (C.N.-l., R.P.), and Shoklo Malaria Research Unit, Mahidol–Oxford Tropical Medicine Research Unit, Faculty of Tropical Medicine, Mahidol University, Mae Sot (C.S.C., F.H.N., G.B.) — both in Thailand; the Centre for Tropical Medicine and Global Health (C.S.C., F.H.N., G.B.) and the Oxford Centre for Clinical Tropical Medicine (B.A.), Nuffield Department of Medicine, University of Oxford, Oxford, GlaxoSmithKline, Stockley Park West (G.C., V.M.R., S.W.J., E.H., D.D.C., K.M., G.C.K.W.K., J.A.G.), and GlaxoSmithKline, Stevenage (L.K.) — all in the United Kingdom; Medicines for Malaria Venture, Geneva (S.D.); and GlaxoSmithKline, Collegeville, PA (V.M.W., J.J.B.).
